# High-throughput mutagenesis reveals functional determinants for DNA targeting by activation-induced deaminase

**DOI:** 10.1093/nar/gku689

**Published:** 2014-07-26

**Authors:** Kiran S. Gajula, Peter J. Huwe, Charlie Y. Mo, Daniel J. Crawford, James T. Stivers, Ravi Radhakrishnan, Rahul M. Kohli

**Affiliations:** 1 Division of Infectious Diseases, Department of Medicine, Perelman School of Medicine, University of Pennsylvania, Philadelphia, PA 19104, USA; 2 Department of Bioengineering, University of Pennsylvania, Philadelphia, PA 19104, USA; 3 Department of Biochemistry and Biophysics, Perelman School of Medicine, University of Pennsylvania, Philadelphia, PA 19104, USA; 4 Department of Pharmacology and Molecular Sciences, Johns Hopkins University School of Medicine, Baltimore, MD 21205, USA

## Abstract

Antibody maturation is a critical immune process governed by the enzyme activation-induced deaminase (AID), a member of the AID/APOBEC DNA deaminase family. AID/APOBEC deaminases preferentially target cytosine within distinct preferred sequence motifs in DNA, with specificity largely conferred by a small 9–11 residue protein loop that differs among family members. Here, we aimed to determine the key functional characteristics of this protein loop in AID and to thereby inform our understanding of the mode of DNA engagement. To this end, we developed a methodology (Sat-Sel-Seq) that couples saturation mutagenesis at each position across the targeting loop, with iterative functional selection and next-generation sequencing. This high-throughput mutational analysis revealed dominant characteristics for residues within the loop and additionally yielded enzymatic variants that enhance deaminase activity. To rationalize these functional requirements, we performed molecular dynamics simulations that suggest that AID and its hyperactive variants can engage DNA in multiple specific modes. These findings align with AID's competing requirements for specificity and flexibility to efficiently drive antibody maturation. Beyond insights into the AID-DNA interface, our Sat-Sel-Seq approach also serves to further expand the repertoire of techniques for deep positional scanning and may find general utility for high-throughput analysis of protein function.

## INTRODUCTION

Enzyme families often share a central well-structured catalytic core, with different specificities among family members encoded by variable regions surrounding the active site core (1,2). This mechanism for fulfilling the need for specialization while maintaining core function is evident in the family of AID/APOBEC cytosine deaminase enzymes, which play an important role in adaptive and innate immunity.

Activation-induced deaminase (AID) is the central B-cell enzyme that governs two diversity-generating reactions that are essential for antibody maturation: somatic hypermutation (SHM) and class switch recombination (CSR) (3). In SHM, deamination of cytosine bases within the variable region of the immunoglobulin (Ig) locus populates the gene with uracil bases. Error-prone repair of these uracil lesions generates variations within antibody complementarity determining regions (CDRs) which can result in enhanced antigen binding and increase the effectiveness of adaptive immune responses. In CSR, deamination can alter the nature of the immune response that follows antigen binding. CSR results from the introduction of uracil lesions into opposite strands of DNA in the switch regions upstream of constant genes. Resolution of the resulting dsDNA breaks juxtaposes the variable region encoding antigen specificity with different constant regions to change the antibody from IgM to an alternative isotype. The related APOBEC3 family enzymes (APOBEC3A-H in the human genome) play a role on the other arm of the immune system, contributing to innate immune responses to retroviruses, such as HIV (4). Targeted deamination of cytosines in viral genomic intermediates can lead to degradation, prevent viral integration or result in highly mutated and non-functional viral genomes (5–8).

As part of their mechanism of targeted deamination, AID/APOBEC enzymes engage cytosine in the context of its neighboring nucleotides within ssDNA. AID prefers to mutate WRC motifs (W = A/T, R = A/G), which are highly populated within both the target CDRs and switch regions in the Ig locus (9). APOBEC3 enzymes are also directed to various hotspot motifs for deamination, with well characterized targeting of CCC by A3G and TC for A3A as examples (5,8,10,11). Targeting of preferred ‘hotspot’ sequences by the deaminases can be essential to their physiological function, as altered hotspot targeting of AID compromises SHM and CSR (12,13). Hotspot targeting has also been key to deciphering the role of APOBEC3 family members in driving mutagenesis in cancerous cells, as the deaminases leave a distinctive mutational signature with clustered mutations enriched for a characteristic TC sequence context (14,15).

Notably, engagement of target sequences by AID/APOBEC enzymes does not show the level of fidelity seen with many other DNA modifying enzymes, such as restriction enzymes. For AID, the differences between deamination of best (hotspot) and worst (coldspot) substrates is ∼12- to 30-fold, raising the question of how such loose specificity can be achieved (16,17). While the lack of a DNA-bound structure for any AID/APOBEC family member leaves many open questions (18–23), structure-guided experiments by several groups have helped to localize some of the determinants for deamination targeting. In particular, one highly divergent 9–11 amino acid protein loop situated between the β4 strand and α4 helix in AID was suggested to be a candidate for conferring sequence preferences to the enzymes (8). In early studies, selective point mutations in this loop altered the spectrum of deaminase activity (24). Even more strikingly, when the loop from one family member was replaced by the loop from a second family member, the sequence targeting of the acceptor enzyme was noted to shift to that of the donor (12,13,17,25).

Given the significance of the hotspot recognition loop in AID and the larger APOBEC family, we aimed to elucidate the specific functional requirements of the residues within this loop. Building on precedents for characterizing deeply mutated proteins, we developed a methodology to efficiently reveal the functional determinants in a small region of a protein by coupling the generation of a library of barcoded saturation mutants, with iterative functional selection and deep sequencing (Sat-Sel-Seq). The method enabled us to perform a comprehensive structure–function analysis of the hotspot recognition loop of AID. By coupling the biochemical analysis to molecular dynamics (MD) simulations, our study helps define the mechanism by which the hotspot loop governs engagement of AID with its DNA substrate.

## MATERIALS AND METHODS

### Cloning, expression and purification of human AID

Synthetic oligonucleotides for cloning and assays were purchased from Integrated DNA Technologies and sequences are available upon request. Alanine scanning loop variants were generated by overlap extension polymerase chain reaction (PCR) as previously described (17). Inserts were cloned into the EcoRI-XhoI region of AID-expressing pET41b vector (Novagen) also containing an N-terminal maltose-binding protein, with the human AID gene codon optimized for expression in *Escherichia coli* as previously described (17). Plasmids were co-transformed with the chaperone trigger factor for heterologous expression in *E. coli* BL21 (DE3) pLysS (Novagen). Enzyme expression and purification were carried out as previously described (26).

### Deamination assays and sequence preference profiles

Similar to previously described protocols (17), the substrate was a 27-mer oligonucleotide containing a single C within an AGC context. For fluorescence-based assays, 1 μM substrate containing a 3′-fluorescein was incubated with 1 μM enzyme, 1 unit uracil DNA glycosylase (UDG; NEB) and 1 μg RNase A (Fermentas) in 20 mM Tris-Cl (pH 8), 1 mM dithiothreitol (DTT), 1 mM ethylenediaminetetraacetic acid (EDTA) for 3 h at 30°C followed by heating to 95°C for 20 min. For kinetic assays, the substrate was ^32^P end-labeled by standard methods, gel purified and quantified using liquid scintillation counting. An end-labeled 40-mer was also generated as an internal standard. In the assay, 50–300 nM radioactive substrate was incubated with 15 nM standard oligonucleotide under reaction conditions described above for 1 h at 30°C (within linear product formation range). Abasic sites formed in the substrates were cleaved by adding NaOH (100 mM final), an equal volume of formamide and heating to 95°C for 20 min. Samples were then separated on 20% Tris/Borate/EDTA (TBE), 7 M Urea polyacrylamide gels (45–50°C). For assays with fluorescent substrate, gels were imaged using a Typhoon imager (GE healthcare) and the products quantified using QuantityOne (Biorad). For kinetic assays, gels were imaged via storage phosphor screen on the Typhoon imager, quantified using custom MATLAB software, and the total amount of deamination was calculated using the known concentration of the standard oligonucleotide as the reference. Data were fit to the Michaelis–Menten equation using least squares fitting with PRISM (Graphpad) software.

Sequence preference profiles were calculated as previously described (27). Briefly, purified AID-WT, R119G or cvBEST were assayed against an array of 16 substrates containing cytosine in an XXC sequence context, where X = A, 5-methylcytosine, G or T. A total of 150 nM of each substrate was incubated with a fixed amount of enzyme as detailed. Product formation was averaged across substrates sharing the same nucleotide at the -1 or -2 position and the relative reactivity for different nucleotides was used to derive the sequence preference.

Rifampicin mutagenesis assays were carried out as previously described (17). Briefly, *E. coli* BL21 (DE3) pLysS were transformed with the AID expression plasmid and a pETcoco2 (Novagen) plasmid expressing uracil DNA-glycosylase inhibitor (UGI), hereafter called the selection strain. Overnight cultures grown from single colonies were diluted to an A_600_of 0.3 and grown for 1 h at 37°C before inducing them with 1 mM isopropyl β-D-1-thiogalactopyranoside (IPTG). After 4 h of additional growth, aliquots of cultures were separately plated on Luria Bertani (LB) agar plates containing Rifampicin (100 μg/ml) and plasmid-selective antibiotics. The mutation frequencies were then calculated by the ratio of rifampicin resistant colonies to total population.

### Saturation mutagenesis, selection and sequencing (Sat-Sel-Seq)

The parent vector for generation of the saturation mutant libraries was made to contain AscI and AatII restriction sites flanking the region of interest and a stop codon for added negative selection. For each mutant, a dsDNA oligonucleotide cassette was generated using oligonucleotides which contained 5′ overhangs of MluI (AscI compatible) and AatII sites, the NNS degenerate codon and a silent mutation (positional barcode) immediately 3′ to the NNS codon. The oligonucleotides were 5′-phosphorylated, annealed and ligated into the AscI/AatII digested parent vector. Ligations were transformed into high-efficiency competent cells (NEB 10β), after which 1/10th volume of cells were plated to determine the library size, while the rest were used to inoculate 25 ml of LB/Kanamycin and grown at 37°C prior to plasmid extraction, resulting in the G0 library at each position. The library sizes were all >100-fold represented and the presence of the degenerate NNS codon in the library was verified by Sanger sequencing.

The plasmid libraries were transformed into the selection strain and 1/10 of the culture was plated to verify the library size. The remaining liquid culture was grown overnight and diluted into 10 independent cultures at A_600_ of 0.3. Cultures were grown for 1 h at 37°C, induced with 1 mM IPTG and after 3 h of additional growth 1 ml of each culture was plated on LB agar with rifampin (100 μg/ml). The rifampin resistant colonies on each plate were washed with 5 ml media and the pooled 50 ml culture with LB Kanamycin was grown overnight. Selection across generations always maintained at least 10-fold overrepresentation of the library. The extracted plasmid encoded the next-generation library, which could be transformed into a naïve selection strain to restart the selection cycle.

The region in AID spanning nucleotides 211–507 was PCR amplified using one of four primer sets that distinguished G0, G1, G2 and G3. From the 5′-direction forward, PCR primers contained a leader sequence for 454 sequencing, an 8 bp DNA barcode (different for each generation) and the touchdown sequence for AID amplification. The PCR products were gel purified and the 48 samples (12 positions × 4 generations) were pooled in equal amounts. A total of 2.5 μg of DNA was subjected to high-throughput sequencing on a Roche 454 GS FLX sequencer (DNA Sequencing Facility, University of Pennsylvania). As depicted in Supplementary Figure S1, the aligned sequence reads were filtered to remove sequence lacking either the generational barcode or a single positional barcode and then each codon identity from each read at the variable position was tabulated.

Selection by covariation of loop residues was performed by constructing eight different sublibraries using oligonucleotides as shown in Supplementary Figure S2. These were pooled in the ratio of 2:2:2:2:1:1:1:1, respectively, to generate the starting library that contained equal amount of each of the 384-library family members. The library was then subjected to several rounds of rifampicin selection as described in Sat-Sel-Seq method above. At the conclusion of selection, 28 individual colonies were sequenced and cataloged.

### MD modeling

Modeller 2.0 (28) was used to generate 1000 homology model structures of AID (1–181) using the crystal structure of A3G (PDB 3IQS) (21), which has 46% sequence identity, as a template. Residue-by-residue energy profiles generated by the discrete optimized protein energy statistical potential (29) was used to analyze the generated models and select a structure showing best fit between the target and template (Supplementary Figure S3A). The model was further refined with extensive MD simulations prior to data collection. The model was equilibrated by constructing an ionized water box (see below) around the protein and subjecting it to a 40 ns constant volume and temperature (NVT) MD simulation (details below). A four base, single-stranded DNA segment was manually modeled in the structure based on the published models of APOBEC3A (30). The AID-WT/ssDNA structure was subjected to an additional 15 ns of NVT MD simulations. At this point, the nucleobase sequence was altered to the favored and disfavored substrate, ensuring that the starting models have a root mean square deviation (RMSD) of 0 Å (Supplementary Figure S3B).

Visual MD (VMD) was used to prepare systems for simulation (31). The VMD Mutator Plugin (Version 1.3) was used to generate Y114F, R119G and cvBEST mutant structures. The structures were solvated with the VMD Solvate Plugin (Version 1.5) with 12 Å of TIP3P H_2_O padding. Each system was ionized and neutralized using the VMD Autoionize Plugin (Version 1.3) to randomly place 0.15 M Na^+^ and Cl^−^ ions with a minimum distance of 5 Å between ions and protein or any two ions. All MD simulations were performed using NAMD program (Version 2.8) with the CHARMM27 force field parameters (32,33). Periodic boundary conditions were used throughout the simulations. Long-range electrostatic interactions were treated with the particle mesh Ewald algorithm (34). Rigid waters were constrained with the SETTLE algorithm (35) and all other constraints were treated with the RATTLE algorithm (36). Bonds between hydrogens and heavy atoms were constrained to their equilibrium lengths. A smooth switching function at 10 Å with a cut-off distance of 12 Å was applied to long-range Van der Waals’ forces. An integration time step of 2 fs was chosen.

A conjugate gradient energy minimization was applied to the solvated, ionized systems before the systems were gradually heated to 300 K. The volume of the solvation box was equilibrated with constant temperature and pressure simulations at 300 K and 1 atm using a Nosé–Hoover Langevin piston (37,38). Harmonic constraints were applied to the N4 atom of the target cytosine, OD1 atom of D89, and the active site Zn^2+^ ion for 40 ns of NVT trajectory. After the initial 40 ns equilibration, harmonic constraints on the cytosine were released, and the simulation was carried out for an additional 120 ns (for a total of 160 ns). All analyses were only performed on the final 120 ns of NVT trajectory with unconstrained DNA.

Hydrogen bond occupancy analysis and solvent accessible surface area were computed as noted in supplementary tables. Contact analysis was performed using the residueDistanceMatrix function implemented in the TCL-VMD distance matrix utilities (Version 1.3), which measures the minimum distance between atomic centers in protein residues to DNA atoms.

## RESULTS

### Alanine scanning mutagenesis

By swapping segments between family members, prior studies have isolated the key determinants of hotspot recognition to a narrow region within human AID, spanning Leu113-Pro123 (12,17,25). In order to understand the molecular basis for the function of this loop, we first employed classical alanine scanning, mutating each amino acid position to alanine (or in the case of Ala121 generating A121G). We focused our efforts on AID expressed with deletion of the C-terminal exon (an 18 amino acid truncation, referred to as AID-WT hereafter for clarity and ease of comparison to mutants), and expressed it as an N-terminal MBP fusion protein for *in vitro* assays. C-terminal truncations of AID have previously been shown result in enhanced solubility for *in vitro* assays and enhanced deaminase activity, making for a larger dynamic range for analysis of mutants, without altering hotspot targeting (17,26,39–41).

Two well-established complementary assays were used to measure deaminase activity. In a bacterial cell-based assay, overexpression of AID in a cell line that co-expresses a protein inhibitor of uracil DNA glycosylase (UGI), results in an increased frequency of genomic transition mutations. C→T or G→A transition mutations in *rpoB*, the gene encoding RNA polymerase B, can confer resistance to the antibiotic rifampin and fluctuation analysis can be employed to calculate the mutational frequency as a function of population size (42). In the complementary *in vitro* deamination assay, purified AID mutants are reacted with fluorescent end-labeled ssDNA substrates containing a single C in an AGC hotspot sequence context. Products with the target cytosine converted to uracil can then be detected by treatment with UDG followed by alkali-induced fragmentation of the resulting abasic site.

**Figure 1.**
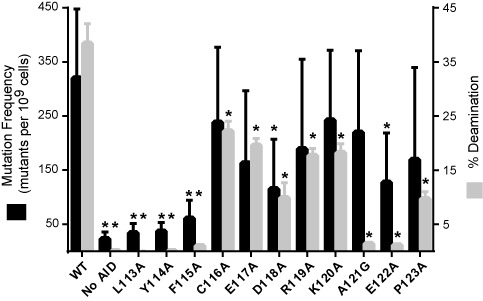
Alanine scanning mutagenesis. The frequencies of acquired rifampin resistance upon expression of AID-WT or variants are given (left axis), with mean (black bars) and standard deviation depicted from at least five independent experiments. The percent product formation under standard *in vitro* assay conditions (1 μM ssDNA S27-AGC substrate, 1 μM enzyme, 3 h incubation) for purified MBP-AID or mutant variants (right axis) are given, with mean (gray bars) and standard deviation from three replicates. No-AID controls are the vector-only control for rifampin assay or the absence of enzyme for the *in vitro* assay. Experimental data that differ from AID-WT with *P* < 0.05 are highlighted with an asterisk (*).

The rifampin mutagenesis assay and the *in vitro* deamination assay demonstrated similar activity patterns in alanine scanning mutagenesis (Figure 1). Within the loop, the N-terminal residues Leu113, Tyr114 and Phe115 appeared essential in both the assays, with activities comparable to the negative controls. The central residues spanning Cys116 to Lys120, along with Pro123, were generally tolerant of alanine mutations, though all showed decreased activity relative to AID-WT. Both the A121G and E122A mutants showed decreased activity relative to AID-WT, although curiously this manifests to a greater extent with the *in vitro* assay than with the rifampin-based bacterial assay. While the patterns are consistent, the differences in the assays points to the importance of using complementary assays to measure deaminase function. Differences could either be related to cellular factors altering protein activity in the rifampin assay or to altered *in vitro* properties of purified deaminases, such as aggregation. Taken together, the consensus of the two approaches suggested the essentiality of the N-terminal region, with more flexibility in the central and C-terminal regions of the protein loop.

### Sat-Sel-Seq for high-throughput analysis of hotspot recognition loop

While alanine scanning mutagenesis assisted in generally localizing functionally important residues, the data failed to reveal a detailed molecular picture of essential and alterable residues in the loop. We, therefore, next developed a method for high-throughput structure–function analysis of the targeting loop. Deep mutational scanning generally involves generation of combinatorial libraries focused on the introduction of random or specific mutations into regions within a target protein (43). Selection for function can then be applied to filter out the poorly active mutants, thus enriching for beneficial mutations. Finally, high-throughput sequencing can quantify the abundance of each variant in the input library as well as in the subsequent libraries obtained after various selection rounds (43,44).

**Figure 2.**
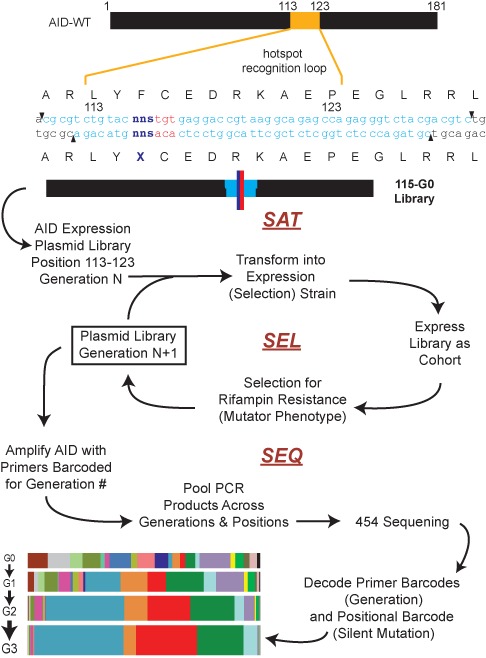
Sat-Sel-Seq methodology. Oligonucleotide cassettes (light blue) containing degenerate codons (NNS) at each position (dark blue) and neighboring silent mutations (red) were cloned into AID expression plasmid to construct saturation mutant libraries. Shown is the representative sequence of a Generation 0 (G0) library for Position 115. The G0 libraries were transformed into *E. coli*, expressed as a cohort and subjected to rounds of selection for acquired resistance to rifampin to generate subsequent generations of the library. The generational libraries were PCR amplified with primers barcoded for the generation number and the pooled PCR samples were sequenced by 454 high-throughput sequencing. The resulting data were decoded using generational and positional bar codes to catalog the evolution of codons over the generations at each position as shown in Supplementary Figure S1.

We designed our method to specifically reveal the determinants of function within a small region of a larger protein (Figure 2). Our strategy for generation of the initial saturation mutagenesis libraries employed a cassette mutagenesis approach (45), which allows for high-efficiency library generation. The mutagenic cassette oligonucleotides contained two key features: a degenerate NNS codon at a single position within the targeting loop of AID and a second silent mutation at the codon immediately 3′ to the randomized codon. This silent mutation serves as an internal barcode that remains unchanged and marks the position of the original NNS codon. Twelve total saturation mutant libraries were generated, one for each position within the hotspot loop and a duplicate library for Phe115 using a different silent mutation barcode in order to assess assay reproducibility. The NNS codon renders each library inclusive of all 20 amino acids and a single stop codon, with all amino acids represented by at least one non-rare codon in *E. coli*. While the library is not equally represented for these variants, the use of an NNS codon allows for economical mutagenesis, and the change in codon frequency can be tracked across generations of selection.

The rifampin mutagenesis assay offers the ability to couple functional enzymatic activity to selection. We introduced our initial Generation 0 (G0) libraries into the selection strain. After inducing expression of the library of AID variants, rifampin resistant colonies were isolated, preserving at least a 10-fold overrepresentation of the library. The AID expression plasmids were recovered from the pooled resistant colonies resulting in the Generation 1 (G1) library. A portion of the library was then transformed into a naïve selection strain and selection for rifampin resistance was iterated over multiple cycles. Based on pilot screening, we saw that highly restrictive positions became fixed by the end of three cycles of selection and we therefore generated plasmid libraries from G0 through G3 at each position.

**Figure 3.**
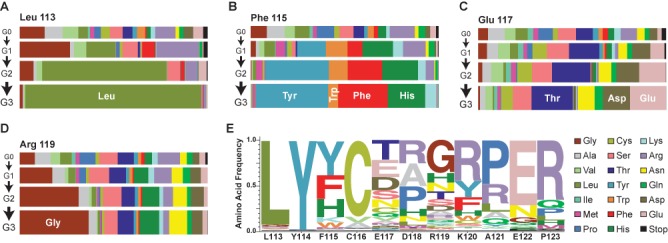
Functional determinants for the Leu113-Pro123 loop revealed by Sat-Sel-Seq. (**A**) Horizontal bar plots depicting the prevalence of amino acids from the four generational libraries, G0–G3. At position Leu113, stringent selection occurs in favor of the wild type (WT) residue. Alternative patterns of selection are evident at (**B**) position Phe115, where aromatic residues are selected by G3, (**C**) position Glu117 that remains highly diversified after selection or (**D**) position Arg119, where mutant variant R119G outcompetes the WT residue over generations. The individual data from other positions are provided in Supplementary Figure S4A. (**E**) Normalized logo plot for residues 113–123 summarizing the frequency of each amino acid at each position in the final selected library. An alternative representative of the G3 data scaled relative to the frequency of each amino acid in the G0 population is provided in Supplementary Figure S4B.

To analyze the impact of selection on the saturation mutant libraries at each position we used barcoded PCR primers, specific to the generation number, to amplify the region of the AID gene centered around the loop. The PCR reaction products (4 generations × 12 positional libraries) were pooled and analyzed in a single run using 454 pyrosequencing. The reads were analyzed for the presence of two barcodes—one in the primers corresponding to the generation number and the second within the loop region encoding the identity of original diversified position (Supplementary Figure S1). Within each bin, the codons at the diversified position were cataloged and the overall frequency of each member was tracked across the generations (Figure 3 and Supplementary Figure S4). Mutations outside of the expected variable positions occurred at a low rate and did not change across generations (0.07% per nucleobase read). Notably, the two saturation mutant libraries at Phe115 that independently underwent selection show a high degree of concordance based upon two different metrics for multivariate similarity, the Euclidean distance and cosine similarity measures (Supplementary Figure S5), demonstrating the assay's reproducibility.

Distinctive patterns of selection appeared that revealed the functional requirements within the loop. Several positions evolved toward their wild-type residue over the selection cycles, notably Leu113 (Figure 3A), Tyr114, Cys116 and Glu122. In these selections, by G3, Leu113 and Tyr114 are both >90% wild type in all reads and Cys116 (84%) and Glu122 (72%) also trend toward fixation (Supplementary Figure S4A). The Phe115 position shows a second pattern, where the degenerate codon evolves to Tyr, Phe, His and Trp in order of decreasing frequency, suggesting the importance of the shared aromatic character for residues at position 115 (Figure 3B). For each of these positions there was general concordance with the results from alanine scanning mutagenesis.

At other positions, alternative distinctive patterns emerged involving evolution away from the native residue. At most of these positions, variability remains high, but a trend toward selection can be observed over generations. For example, at Glu117, polar residues were favored, but the variability remained high after three cycles of selection, suggesting that this is a tolerant position (Figure 3C). In the case of Arg119, each successive generation saw an expansion of a non-native Gly residue (Figure 3D). Since we know that the wild-type residue is tolerated at each of these positions, the rate of evolution away from the native residue points to the extent to which particular mutations may outcompete the wild-type sequence.

To provide an integrated picture of the selection at each position, the distribution of each amino acid in G3 was weighted at each position and expressed as a logo plot (Figure 3E). In agreement with alanine scanning mutagenesis data, the N-terminal residues were less tolerant to alteration than the C-terminal residues. In the C-terminal end of the loop, multiple positions trended toward Arg. This alteration could reflect the higher abundance of Arg in the starting saturation mutant library (three of the NNS codons encode Arg) or arise from enhanced DNA electrostatic interactions. When the logo diagram was corrected for the distribution of amino acids in the starting population (Supplementary Figure S4B), the preference for Arg diminished at many positions, but it remained an enriched residue.

### Selection validation, loop residue covariation and target sequence specificity

**Figure 4.**
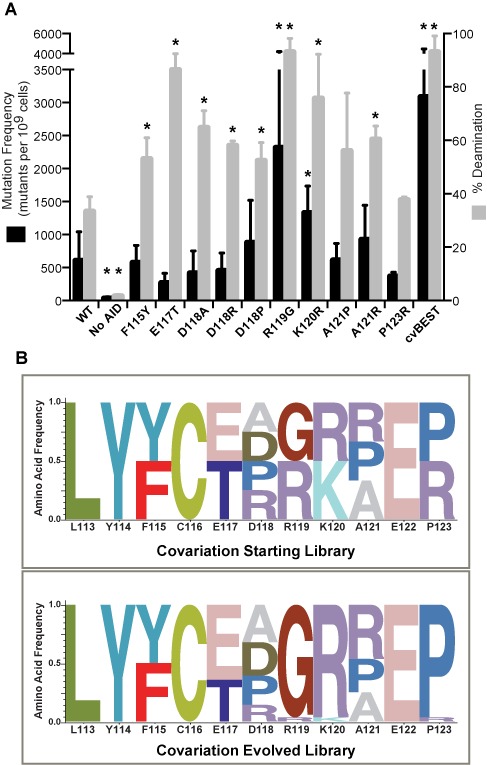
Characterization of selected AID variants. (**A**) Amino acids which evolved to be greater than 20% of the total G3 library at each position were characterized for their activity using the rifampin mutagenesis assay and the *in vitro* deamination assay. The data are represented as in Figure 1. Experimental data that are significantly different from the AID-WT (*P* < 0.05) are highlighted with an asterisk (*). At the right of the plot are shown the results for cvBEST, which was selected based on covariation of the best amino acids at each position as described below. (**B**) To examine the impact of covariation of multiple positions in the loop, a starting pooled library was generated containing the WT amino acid at each position along with any other amino acid that resulted in >20% of the counts in G3 at that position. The covariation library underwent six cycles of selection and at the conclusion of selection, individual colonies were sequenced and the frequency of each amino acid variant was cataloged. The results are summarized as a normalized logo plot on bottom panel.

We next aimed to understand why certain mutations were preferred in the Sat-Sel-Seq procedure. Across all positions, we selected mutants that represented >20% of the total count in G3 and evaluated these mutants in the context of our two complementary deaminase assays. In the rifampin assay, the majority of selected variants had activity equivalent to or greater than AID-WT, within the limits of statistical significance (Figure 4A). When the individual point mutants were purified and evaluated using the *in vitro* deamination assay, all of the variants that were favored over AID-WT in Sat-Sel-Seq showed increased deaminase activity. Among the variants, one notable mutation, R119G, demonstrated the largest enhancement in activity in both the rifampin assay (∼4-fold) and the *in vitro* enzymatic assay (at least 3-fold).

With the benefit of having narrowed the list of tolerated mutations at each position in the targeting loop, we next evaluated the effect of covariation of residues. To that end, we generated a plasmid library containing all preferred residues (>20% from G3 library) as well as wild-type residues (even when these residues were not >20% in G3). The library contained an equal proportion of 384 different variants (Supplementary Figure S2) and was at least 10-fold overrepresented in the starting population. The library was subjected to several rounds of rifampin-resistance-based selection. After six rounds of selection, the pooled plasmid population appeared static as judged by sequencing of the pooled plasmid library and individual colonies were sequenced to determine the distribution of mutations (Figure 4B). Several positions remained heterogeneous after these rounds of selection, including F115F/Y, E117E/T, D118D/A/R/P and A121A/R/P. One of the positions was found to deviate from Sat-Sel-Seq results, where WT Pro123 consistently outcompeted P123R in the covariant selection. Most strikingly, two positions that emerged in Sat-Sel-Seq, R119G and K120R, again showed a clear shift away from the WT residue and emerged as critical alterations in the co-variation analysis.

**Figure 5.**
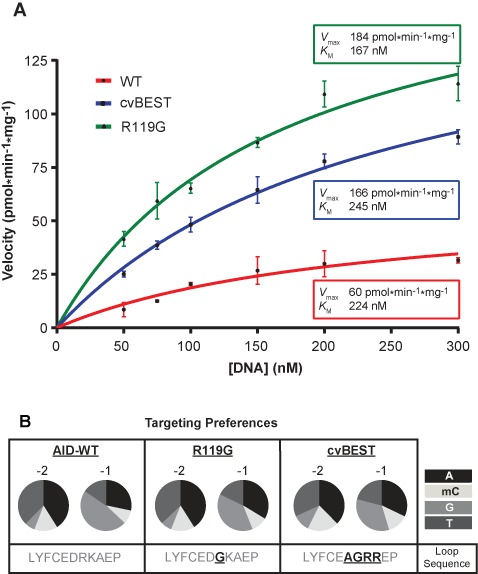
Characterization of hyperactive AID variants. (**A**) Kinetics for deamination with hyperactive AID variants. Deamination assays were carried out with ^32^P end-labeled 27-mer substrate containing a single cytosine in an AGC hotspot. The rate of product formation was determined at various substrate concentrations and the mean and standard deviation from at least three replicates are shown for each condition. The curves were fit to the Michaelis–Menten equation and the determined values for *V*_max_ and *K*_m_ are reported. (**B**) AID-WT, R119G and cvBEST were assayed against an array of substrates that contained a single cytosine in 1 of 16 XXC sequence contexts, where *X* = A, mC, G or T. The percent product formation for each substrate was determined and the overall preferences at the -2 and -1 positions were determined based on the averages across each position (Supplementary Figure S6). The preferences at the -2 and -1 positions are represented as a pie chart for A, mC, G or T.

As our method selects for enhanced deaminase activity, we chose two AID variants for additional detailed analysis: the R119G point mutant and the sequence selected in the covariation experiment (cvBEST, D118A/R119G/K120R/A121R). In both the rifampin mutagenesis assay and in the *in vitro* deamination assay, cvBEST showed enhanced activity relative to AID-WT (Figure 4A). The majority of these enhancements were attributable to the R119G mutation, although some additional enhancement did arise from the mutations in cvBEST. Steady-state kinetic analysis revealed an enhancement in *V*_max_ for the mutant variants relative to AID-WT, with roughly similar *K*_m_ values (Figure 5A). The rate determining step in deamination by AID has not been established and thus mutations could alter substrate binding, the chemical steps in catalysis or product release. Importantly, the agreement between the rifampin assay and *in vitro* experiments suggest that the same step is impacted in both settings.

The R119G and cvBEST hyperactive variants were next tested for their ability to target DNA in different sequence contexts. Using the *in vitro* deamination assay, AID and the hyperactive variants were assayed against 16 related oligonucleotide substrates containing cytosine in different XXC sequence contexts (*X* = A, 5-methylcytosine, G, T) (17). The relative rates of product formation for each substrate were measured and used to assess the sequence targeting specificity. 5-methylcytosine serves as a less reactive surrogate for cytosine and the assay, which cleanly reports on deamination at the target cytosine, has been previously validated to accurately predict the *in vivo* targeting profile of AID variants (13,17,40). While our Sat-Sel-Seq method could have resulted in variants with altered sequence preference, the results instead demonstrate that these hyperactive variants R119G and cvBEST retain a preference for WRC sequences (Figure 5B and Supplementary Figure S6). Preserved targeting suggests the possibility that WRC recognition by AID could take place through multiple modes and that mutations in the targeting loop could specifically promote particular binding modes.

### MD modeling of AID-DNA interactions

The structure of AID remains unsolved and the interactions between AID and its DNA substrate remain a point of conjecture based upon unliganded structures of related APOBEC3 family members (18–23). In an effort to provide a mechanistic explanation of the selection data from the Sat-Sel-Seq, we generated a homology model of AID-WT using the crystal structure of A3G as the protein template (21) and docked a tetranucleotide DNA fragment anchoring the target cytosine in association with the active site Glu58 (Supplementary Figure S3). Several models of AID and the DNA were constructed. These included (i) AID-WT with its hotspot and coldspot DNA substrates, and (ii) the mutants Y114F, R119G and cvBEST each with the hotspot substrate. Each of these DNA-enzyme complex models were subjected to MD simulations, and the final 120 ns of each trajectory was analyzed.

**Figure 6.**
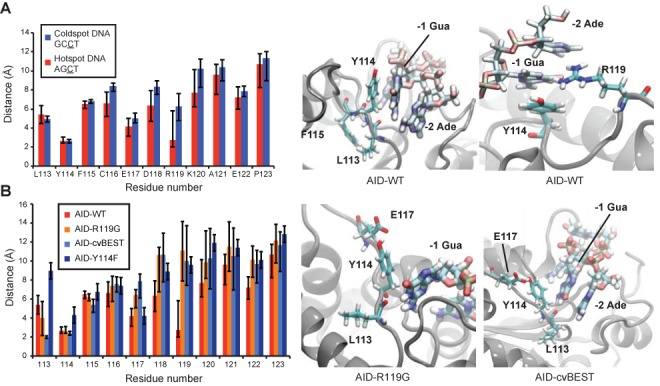
MD simulations of AID interactions with DNA. (**A**) Residue-to-DNA time-averaged distances between targeting loop residues and the hotspot or coldspot DNA sequences. Representative images showing the interactions of Leu113, Tyr114, Phe115 and Arg119 to the hotspot DNA are depicted in the two panels at right. (**B**) Residue-to-DNA time-averaged distances between the AID-WT, Y114F, R119G or cvBEST loops and hotspot DNA, calculated from the sampled distances in the 120 ns simulation trajectories. Notably, the Leu113 backbone oxygen forms closer interactions in the R119G and cvBEST mutants. Snapshots of R119G and cvBEST interacting with the hotspot DNA are shown in the right two panels.

MD simulations of the hotspot (AGCT) (Supplementary Video S1) and coldspot (GCCT) substrate complexes with AID-WT revealed differences in specific protein-DNA contacts. For this modeling, the underlying hypothesis is that perturbed interactions between a specific protein residue and DNA nucleotides result in reduced deaminase activity. Within this analytical framework, the distribution of residue-to-DNA time-averaged distances revealed that WT residues Tyr114 and Arg119 make consistent contacts with the hotspot substrate (Figure 6A). Conversely, only Tyr114 was found to consistently contact the coldspot substrate. With the hotspot substrate, Tyr114 formed aromatic stacking interactions with -1 Guanine throughout the trajectory, and occasionally wedged between the -1 Gua and -2 Ade. Arg119 formed significant hydrogen bonding interactions with -1 Gua N7/O6 and more transient electrostatic interactions with the phosphate linkage between -1 Gua and -2 Ade (Supplementary Table S1). The side chains of residues Leu113 and Phe115 are buried (Supplementary Table S2) and form hydrophobic contacts with one another that shape the surrounding protein architecture, positioning Tyr114 for stacking interactions and the backbone amide of Leu113 for potential hydrogen bonding interactions with the DNA. It should be noted that our unconstrained *in silico* tetranucleotide substrate displayed greater dynamics than might be expected with longer physiological substrates that would be constrained by both the upstream and downstream DNA. Although this presented the challenge of potentially destabilizing some intermolecular interactions, it also conferred the advantage of allowing for greater exploration of conformations and binding poses.

To specifically evaluate the importance of the Tyr114 residue, we additionally simulated the Y114F mutant bound to the preferred hotspot substrate. Based on residue-to-DNA distances, substrate binding was much more robust with the native tyrosine as compared to the Y114F mutant (Figure 6B). Interestingly, the simulations reveal that the hydroxyl group does not make critical specific DNA contacts. Rather, the Tyr hydroxyl promotes transient solvent interactions that prevent the side chain from becoming buried and thereby permit stacking interactions with the -1 Gua and -2 Ade. Tyr114 is only 44.4% buried in AID-WT, while the Y114F residue is 61.6% buried (Supplementary Table S2).

We next evaluated the DNA substrate interactions of two AID variants with enhanced deamination activity, R119G and cvBEST. Although contacts between Arg119 and DNA were abolished as a result of the mutations, the backbone carbonyl oxygen of residue Leu113 now showed increased hydrogen bonding with N1/N2 of the -1 Gua in both models (Figure 6B). This hydrogen bond had occupancy of 0.2% in WT, 22.6% in R119G and 73.7% in cvBEST (Supplementary Table S1). Thus, in these variants with enhanced activity, MD simulations suggest that removing one mode of substrate binding observed in the WT simulation results in a compensatory mode of substrate engagement.

## DISCUSSION

In this work, we have performed high-throughput mutagenesis on a targeted region of the B-cell mutator AID to gain insight into enzyme's targeting mechanism. While prior biochemical studies have highlighted the importance of a key protein loop in targeting (12,13,17,24–26,46,47), the functional requirements for this loop have remained unclear and, despite numerous available structures of AID/APOBEC family members (18–23), no structures yet exist with bound nucleic acid. We turned to high-throughput mutagenesis experimental methods commonly utilized in enzyme engineering and adapted these methods to explore the enigmatic interface between AID and its nucleic acid substrates. The data reveal the modes for DNA substrate engagement and the biochemical insights can be rationalized by molecular dynamic simulations.

The results indicate that the N-terminal segment of the targeting loop is required for deaminase function. Beginning at the N-terminal end of the targeting loop, the wild-type residue Leu113 was highly selected in Sat-Sel-Seq, shows high occupancy in MD simulations with the hotspot substrate and enhanced hydrogen bonding with the -1 Gua in MD simulations of the hyperactive R119G and cvBEST variants (Figure 6). Our MD simulations suggest that this buried side chain can contribute to shaping active site architecture in concert with Phe115 and its importance is further supported by its high conservation (Leu or Ile) across the AID/APOBEC deaminase family. The adjacent residue at Position 114 also shows selective drive toward the wild-type Tyr residue in Sat-Sel-Seq and is fittingly highly conserved across the family. The MD simulations suggest that Tyr114 stacks with the -1 residue of the target sequence and that the preference for Tyr over Phe results from solvent interactions that prevent the side chain's burial (Figure 6B). Finally, in Sat-Sel-Seq, Phe115 evolved to any aromatic residue (Tyr, Trp, His). This aligns well with its role as a buried aromatic residue that can engage in hydrophobic interactions with Leu113 to shape the active site. Notably, our discovery of the requirement for aromatic character at Phe115 is a clear example of the insights attainable through deep mutagenesis in Sat-Sel-Seq that would not be revealed by a conventional Ala scanning mutagenesis approach. Taken together, the residues spanning Leu113-Phe115 form an important and largely immutable scaffold for AID to engage with its substrate.

More diverse modes of DNA recognition are apparent in the loop positions downstream from the N-terminal region. One of the most interesting interactions originates from Arg119. In MD simulations Arg119 is highly engaged with hotspot -1 residue, which seemed contradictory to the enhanced deamination activity of the R119G mutant. However, this increased activity was reasonably accounted for in simulations of the R119G and cvBEST variants where mutation to a glycine allowed for enhanced interactions between the backbone amide carbonyl of Leu113 and the -1 purine. Notably, this interaction would not be possible with a smaller pyrimidine. The biochemical analysis and MD simulations of the hyperactive R119G and cvBEST variants suggest that multiple potential binding modes can result in the recognition of the preferred hotspot sequences (WRC) (Figure 5B). Importantly, the MD simulations suggest that the alternative binding modes do not explicitly involve the mutated residues, raising the possibility that these modes, though less favored than the recognition mode involving Arg119, could potentially be accessed by the wild-type enzyme as well. The maintenance of WRC recognition despite mutations in the targeting loop are in line with a separate study examining zebrafish AID that concluded that the overall loop architecture and its flexibility, as opposed to specific residues, were important for the enzyme's ability to target 5-methylcytosine for deamination (48). Our finding of relative tolerance in the targeting loop from AID stands in contrast to a study on A3G where a single point mutation was able to convert the enzyme from preferred targeting of CC to TC hotspot motifs and similar findings with A3F (19,46). Notably, AID is distinguished from its APOBEC3 relatives in the size of its recognition loop (11 amino acids versus 9–10 in most APOBEC3 enzymes) and in targeting cytosine following a -1 position purine (as opposed to pyrimidine). While it is possible that additional determinants outside of the loop may contribute to hotspot recognition, these features inherent to AID may explain its distinctive substrate recognition, as well as the fact that point mutations in the targeting loop do not alter the enzyme's selectivity.

In addition to revealing the functional requirements within the targeting loop of AID, our selection method yielded several hyperactive variants. In a prior study, random mutagenesis was coupled to a lac papillation mutagenesis assay to yield hyperactive AID variants which were associated with higher rates of pathological chromosomal translocations (49). Interestingly, in this study two hyperactive mutations localized to the targeting loop (F115Y, K120R). These mutations also emerged as preferred residues in our Sat-Sel-Seq approach. Despite the fact that our approach was directed at the targeting loop only, cvBEST showed a 3-fold enhancement in the *in vitro* deamination rate, as high as the best variants selected through mutagenesis of the entire AID gene (49). This result suggests that the primary determinants for enhancing deamination activity lie in the loop region. While the rate limiting step in deamination is not currently known, it is possible that alterations in either substrate engagement or product release could explain the higher activity observed with R119G or cvBEST. In line with prior work on hyperactive AID (49), we speculate that our hyperactive variants are not commonly found natural polymorphisms, as concerns about genomic stability resulting from higher deamination activity may have constrained AID's evolution.

Our study demonstrates the utility of high-throughput mutagenesis strategies for gaining functional insights into proteins and is worthy of comparison to existing methods (43,50). One prior approach that has been particularly useful for high-throughput mutagenesis is phage display technology, where selection is based on binding (43,51,52). Random chemical mutagenesis coupled to deep sequencing has also been used to select for functional requirements at a genome-wide level (53), and deep mutagenesis coupled to a single round of selection for antibiotic resistance has been used to reveal the functional determinants of β-lactamase (54). Sat-Sel-Seq extends such approaches in several useful manners. In Sat-Sel-Seq, selection is based on enzymatic activity rather than binding, mutagenesis is confined to a key region of a protein, and the process is iterated over multiple generations to reveal the evolutionary time course. While our method can readily be translated to the study of other mutator enzymes or enzymes whose function can be directly tied to survival (e.g. antibiotic resistance elements or toxic genes) (54,55), we speculate that Sat-Sel-Seq could find a role in structure–function relationship in other proteins. The approach could be combined with conventional phage or bacterial surface display, with newer methods in *E. coli* that couple general protein–protein interactions with selectable resistance to antibiotics or with high-throughput screening methods that exploit *in vitro* compartmentalization (56–58). Our work establishes the utility of deep mutagenesis for providing insight into a poorly defined interface between an enzyme and its substrate and should be generalizable to other proteins with small regions that encode critical functional determinants.

Several factors in Sat-Sel-Seq could be optimized to address alternative questions or improve the approach. We chose to construct a library at each position and perform selection in parallel, as this approach yielded equivalent data on selection at each position, rather than allowing a few hyperactive clones to emerge and dominate the population. However, if hyperactivity rather than structure–function analysis was the goal of the experiments, the protocol could be altered by performing pooled selection. Randomization of multiple positions simultaneously could also be used to reveal potential epistatic interactions. Finally, if coupled to alternative next-generation sequencing methods, Sat-Sel-Seq could yield an even larger number of observations on a protein of interest.

In antibody maturation, targeting of WRC hotspot sequences within the Ig locus is essential to proper SHM and CSR, and these sequences are fittingly enriched in CDRs and switch regions (12,13,59). Our biochemical data and MD simulations suggest that DNA targeting can occur in multiple binding modes through the dynamic hotspot recognition loop. Rather than being driven by specific contacts, AID demonstrates looser interactions that can select for preferred substrates, but not to the exclusion of other potential interactions, as evidenced in the alternative binding mode that emerged in the hyperactive variants. Flexible binding modes potentially reflect on the delicate balance between specificity and activity that is required for AID. In line with the hypothesis that diversity is best generated by ‘haphazard’ deamination (60), multiple modes of interacting with DNA substrates could provide a mechanism that increases the scope of antibody diversity while preserving the advantages of targeting CDRs and switch regions. While our studies provide a surrogate molecular level view, further biochemical studies on other deaminase family members, and ultimately high-resolution structural insight into the DNA binding mode of AID/APOBEC deaminases, will be key to resolving how these deaminases can achieve targeted and purposeful mutation of DNA.

## SUPPLEMENTARY DATA

Supplementary Data are available at NAR Online.

SUPPLEMENTARY DATA
