# Supplementary material for: High-throughput mutagenesis reveals functional determinants for DNA targeting by activation-induced deaminase
Source: Nucleic Acids Res. 2014 Jul 26;42(15):9964–75. doi: 10.1093/nar/gku689 (PMC4150791; doi:10.1093/nar/gku689)
Supplement: SUPPLEMENTARY DATA [file supp_42_15_9964__index.html]

High-throughput mutagenesis reveals functional determinants for DNA targeting by activation-induced deaminase — High-throughput mutagenesis reveals functional determinants for DNA targeting by activation-induced deaminase — SUPPLEMENTARY DATA 

# High-throughput mutagenesis reveals functional determinants for DNA targeting by activation-induced deaminase

## SUPPLEMENTARY DATA

**Files in this Data Supplement:**

- SUPPLEMENTARY DATA
- SUPPLEMENTARY DATA
