# Supplementary material for: High-throughput mutagenesis reveals functional determinants for DNA targeting by activation-induced deaminase
Source: Nucleic Acids Res. 2014 Jul 26;42(15):9964–75. doi: 10.1093/nar/gku689 (PMC4150791; doi:10.1093/nar/gku689)
Supplement: SUPPLEMENTARY DATA [file supp_gku689_nar-01532-z-2014-File008.pdf]

High-throughput mutagenesis reveals functional determinants for DNA targeting by Activation-Induced Deaminase

Kiran S. Gajula, Peter J. Huwe, Charlie Y. Mo, Daniel J. Crawford, James T. Stivers, Ravi Radhakrishnan and Rahul M. Kohli

SUPPLEMENTARY INFORMATION

Supplementary Figures S1-S6, Supplementary Tables S1-S2, Supplementary Video S1

SUPPLEMENTARY FIGURES

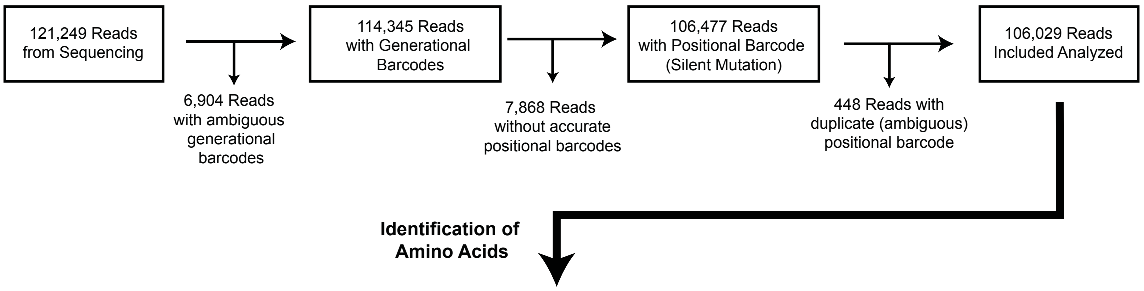

	Gly	Ala	Val	Leu	Ile	Met	Pro	Cys	Ser	Thr	Tyr	Trp	Phe	His	Lys	Arg	Asn	Gln	Asp	Glu	Stop	Total
**113N-G0**	378	270	236	144	34	65	97	136	194	148	66	125	23	60	66	307	57	64	146	150	66	2832
**113N-G1**	573	45	141	332	3	6	38	32	176	22	9	18	148	5	7	490	2	28	5	13	45	2138
**113N-G2**	170	37	68	1517	1	1	1	7	154	3	3	3	49	4	5	148	1	6	4	100	10	2292
**113N-G3**	55	19	20	2941	0	2	3	2	24	6	5	2	7	0	0	32	1	1	0	24	0	3144

	Gly	Ala	Val	Leu	Ile	Met	Pro	Cys	Ser	Thr	Tyr	Trp	Phe	His	Lys	Arg	Asn	Gln	Asp	Glu	Stop	
**114N-G0**	140	162	119	120	23	53	208	78	163	148	92	58	26	64	37	190	47	77	51	56	31	1943
**114N-G1**	145	77	8	12	3	3	746	10	25	25	576	24	5	0	0	71	1	5	211	0	2	1949
**114N-G2**	26	11	2	2	1	0	83	4	12	12	2344	9	2	2	0	100	0	1	60	0	0	2671
**114N-G3**	3	1	0	2	0	1	21	0	0	0	2665	1	0	2	0	3	0	1	8	0	0	2708

	Gly	Ala	Val	Leu	Ile	Met	Pro	Cys	Ser	Thr	Tyr	Trp	Phe	His	Lys	Arg	Asn	Gln	Asp	Glu	Stop	
**115N-G0**	227	250	141	205	46	55	243	71	197	151	48	85	59	115	48	350	53	93	68	83	35	2623
**115N-G1**	73	48	72	151	8	135	26	69	52	22	403	305	211	430	141	319	44	81	21	27	9	2647
**115N-G2**	19	11	16	70	0	136	3	30	14	4	1341	396	726	752	161	183	21	52	3	8	5	3951
**115N-G3**	2	4	6	9	2	28	1	5	4	1	829	114	569	431	127	18	2	3	1	1	1	2158

	Gly	Ala	Val	Leu	Ile	Met	Pro	Cys	Ser	Thr	Tyr	Trp	Phe	His	Lys	Arg	Asn	Gln	Asp	Glu	Stop	
**116N-G0**	319	327	162	281	96	69	333	151	336	290	83	110	69	125	82	505	96	72	108	80	60	3754
**116N-G1**	75	159	121	77	33	21	32	279	18	39	109	25	49	44	10	89	27	6	4	5	2	1224
**116N-G2**	91	211	249	42	19	12	21	1085	14	22	111	11	67	6	8	45	6	0	0	1	0	2021
**116N-G3**	12	115	139	3	5	2	4	1796	3	4	9	5	14	1	2	6	7	0	0	0	0	2127

	Gly	Ala	Val	Leu	Ile	Met	Pro	Cys	Ser	Thr	Tyr	Trp	Phe	His	Lys	Arg	Asn	Gln	Asp	Glu	Stop	
**117N-G0**	213	358	231	259	119	111	308	108	389	359	123	89	113	133	140	350	165	95	177	93	92	4025
**117N-G1**	48	60	65	43	29	21	29	78	120	164	19	10	10	29	17	18	48	27	99	77	8	1019
**117N-G2**	37	80	110	23	46	44	11	133	180	344	7	2	11	33	4	18	114	46	205	239	2	1689
**117N-G3**	21	57	81	16	34	35	6	128	204	448	10	2	10	14	9	13	182	89	297	392	1	2049

	Gly	Ala	Val	Leu	Ile	Met	Pro	Cys	Ser	Thr	Tyr	Trp	Phe	His	Lys	Arg	Asn	Gln	Asp	Glu	Stop	
**118N-G0**	183	226	151	239	78	71	270	70	302	235	65	77	71	109	96	301	109	105	128	80	81	3047
**118N-G1**	11	27	5	85	3	5	231	3	19	13	12	4	3	33	3	239	71	4	13	11	3	798
**118N-G2**	10	372	5	620	2	119	1026	4	17	19	25	0	3	181	4	839	210	6	17	15	5	3499
**118N-G3**	7	834	0	152	0	66	830	2	21	27	8	1	2	262	2	845	195	6	20	24	1	3305

	Gly	Ala	Val	Leu	Ile	Met	Pro	Cys	Ser	Thr	Tyr	Trp	Phe	His	Lys	Arg	Asn	Gln	Asp	Glu	Stop	
**119N-G0**	225	235	141	172	57	59	237	55	273	241	61	47	53	108	86	330	101	87	100	87	34	2789
**119N-G1**	106	41	11	23	1	11	22	9	66	55	10	11	4	47	19	57	36	24	34	22	0	609
**119N-G2**	1055	189	44	80	12	38	26	49	329	238	60	46	26	308	69	175	250	70	231	67	0	3362
**119N-G3**	950	111	12	33	2	17	7	33	183	220	35	28	15	273	54	83	247	62	149	66	3	2583

	Gly	Ala	Val	Leu	Ile	Met	Pro	Cys	Ser	Thr	Tyr	Trp	Phe	His	Lys	Arg	Asn	Gln	Asp	Glu	Stop	
**120N-G0**	180	243	139	218	60	57	326	74	212	169	78	87	52	98	47	335	74	59	71	57	52	2688
**120N-G1**	25	30	10	16	2	11	12	23	31	26	52	23	47	35	8	155	10	12	8	7	5	548
**120N-G2**	32	72	20	51	2	36	20	99	92	37	451	193	324	184	17	1156	71	23	11	26	2	2919
**120N-G3**	7	31	5	14	0	7	6	92	56	25	419	118	345	138	2	1052	11	2	7	2	1	2340

	Gly	Ala	Val	Leu	Ile	Met	Pro	Cys	Ser	Thr	Tyr	Trp	Phe	His	Lys	Arg	Asn	Gln	Asp	Glu	Stop	
**121N-G0**	87	113	82	116	28	22	150	34	111	67	31	36	24	44	18	175	22	44	36	34	34	1308
**121N-G1**	12	36	8	32	3	15	89	3	27	9	0	9	12	7	13	79	11	10	10	8	0	393
**121N-G2**	39	250	28	223	2	121	1251	21	143	8	19	34	19	40	48	513	56	108	28	15	3	2969
**121N-G3**	6	178	42	221	0	13	1166	0	12	1	61	0	0	8	25	515	4	154	2	2	0	2410

	Gly	Ala	Val	Leu	Ile	Met	Pro	Cys	Ser	Thr	Tyr	Trp	Phe	His	Lys	Arg	Asn	Gln	Asp	Glu	Stop	
**122N-G0**	139	142	128	178	26	54	166	72	158	87	29	68	36	41	49	200	32	60	8	73	49	1795
**122N-G1**	119	42	9	10	0	1	15	11	18	8	3	5	25	15	1	10	31	75	1	74	3	476
**122N-G2**	636	279	2	17	0	6	52	121	84	37	6	3	20	55	0	10	582	266	0	1757	7	3940
**122N-G3**	155	57	1	5	0	8	9	30	23	4	0	2	13	11	1	1	353	68	1	1894	2	2638

	Gly	Ala	Val	Leu	Ile	Met	Pro	Cys	Ser	Thr	Tyr	Trp	Phe	His	Lys	Arg	Asn	Gln	Asp	Glu	Stop	
**123N-G0**	88	63	89	153	41	52	72	35	108	41	44	54	45	52	60	170	38	48	41	28	31	1353
**123N-G1**	5	17	6	23	1	3	39	12	13	1	13	4	5	15	6	101	7	17	3	2	0	293
**123N-G2**	20	48	25	114	7	76	280	38	31	9	9	3	2	31	10	1919	15	181	4	7	1	2830
**123N-G3**	2	12	8	142	4	94	207	14	4	1	1	2	2	126	3	1715	12	238	0	2	2	2591

	Gly	Ala	Val	Leu	Ile	Met	Pro	Cys	Ser	Thr	Tyr	Trp	Phe	His	Lys	Arg	Asn	Gln	Asp	Glu	Stop	
**115Alt-G0**	94	46	35	20	3	11	40	19	44	21	16	23	10	16	8	86	15	20	17	19	4	567
**115Alt-G1**	368	38	22	22	2	42	13	23	24	13	96	72	25	105	33	86	4	308	10	7	6	1319
**115Alt-G2**	97	14	6	6	0	72	2	16	6	5	448	130	109	307	36	65	1	282	0	2	2	1606
**115Alt-G3**	22	0	2	1	0	47	2	1	4	0	868	194	182	615	14	31	0	107	0	0	0	2090

Total Reads 106029

Figure S1. Deconvolution of Sequencing Data from Sat-Sel-Seq. Shown is the total number of reads from 454 sequencing, filtered by the presence of barcodes for the generation number, by internal barcodes encoding for position number and eliminating any variants with redundant barcodes. The remaining reads were binned by the two barcodes and the identity of the codon at the mutagenized position was cataloged. The total number of reads for each condition are shown, with the relative frequencies of each amino acid reported in Figure S4.

SL1

		Y			A	G	R	P		R
L	Y	F	C	E	D	R	K	A	E	P
TTA	TAT	TWT	TGC	GAA	GMT	SGG	ARA	SCC	GAG	CSC

SL2

		Y			A	G	R	P		R
L	Y	F	C	T	D	R	K	A	E	P
TTA	TAT	TWT	TGC	ACC	GMT	SGG	ARA	SCC	GAG	CSC

SL3

		Y			R	G	R	P		R
L	Y	F	C	E	P	R	K	A	E	P
TTA	TAT	TWT	TGC	GAA	CSC	SGG	ARA	SCC	GAG	CSC

SL4

		Y			R	G	R	P		R
L	Y	F	C	T	P	R	K	A	E	P
TTA	TAT	TWT	TGC	ACC	CSC	SGG	ARA	SCC	GAG	CSC

SL5

		Y			A	G	R			R
L	Y	F	C	E	D	R	K	R	E	P
TTA	TAT	TWT	TGC	GAA	GMT	SGG	ARA	CGG	GAG	CSC

SL6

		Y			A	G	R			R
L	Y	F	C	T	D	R	K	R	E	P
TTA	TAT	TWT	TGC	ACC	GMT	SGG	ARA	CGG	GAG	CSC

SL7

		Y			R	G	R			R
L	Y	F	C	E	P	R	K	R	E	P
TTA	TAT	TWT	TGC	GAA	CSC	SGG	ARA	CGG	GAG	CSC

SL8

		Y			R	G	R			R
L	Y	F	C	T	P	R	K	R	E	P
TTA	TAT	TWT	TGC	ACC	CSC	SGG	ARA	CGG	GAG	CSC

Figure S2. Sub-libraries for covariation selection. Using the oligonucleotides shown, eight different sub-libraries were created. These when pooled in the ratio of 2:2:2:2:1:1:1:1 generate a starting library that contain equal amount of each of the 384-library family members.

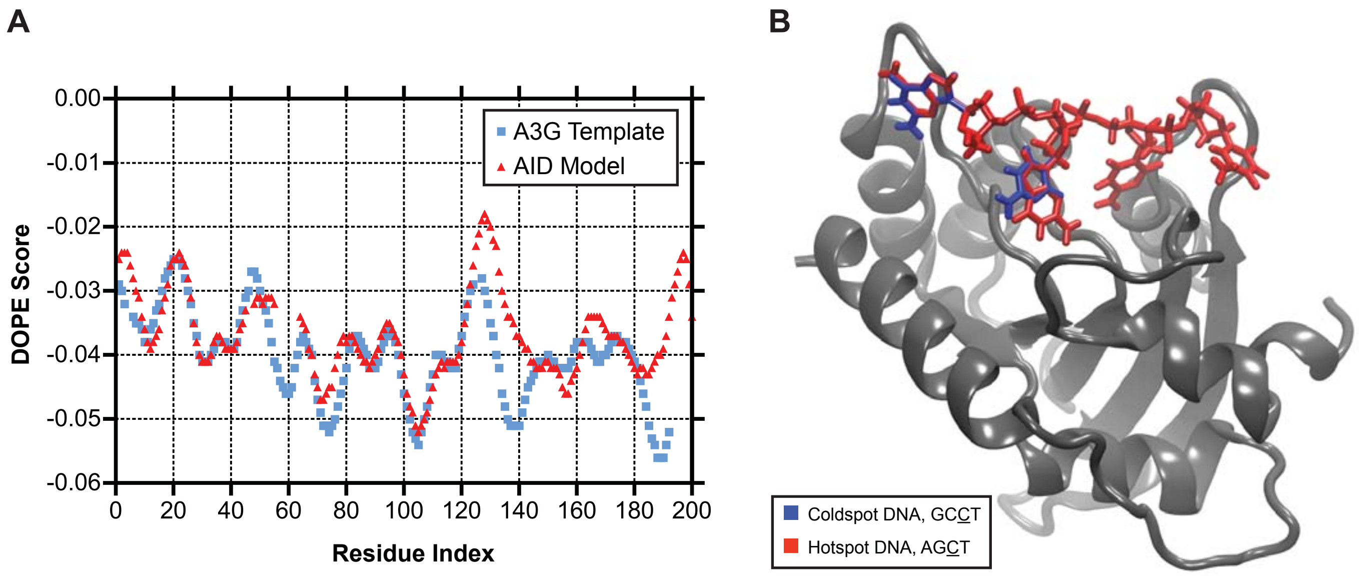

Figure S3. AID Structural Model (**A**) The Discrete Optimized Protein Energy (DOPE) profiles for the template (A3G) and selected target (AID homology) structures are shown. The selected AID homology structure in red showed good fit to the template structure in blue. The selected model was further refined with extensive MD simulations. (**B**) Model of AID bound to DNA. Shown is the homology model of AID(1-181), based on the structure of A3G (PDB 3IQS), with bound ssDNA containing either a hotspot (AGCT) or coldspot (GCCT) sequence, colored red and blue respectively. The alternations in nucleobase composition were done after pre-equilibration of the model, making the starting point for MD simulations identical (RMSD of 0 Å between AID in two structures).

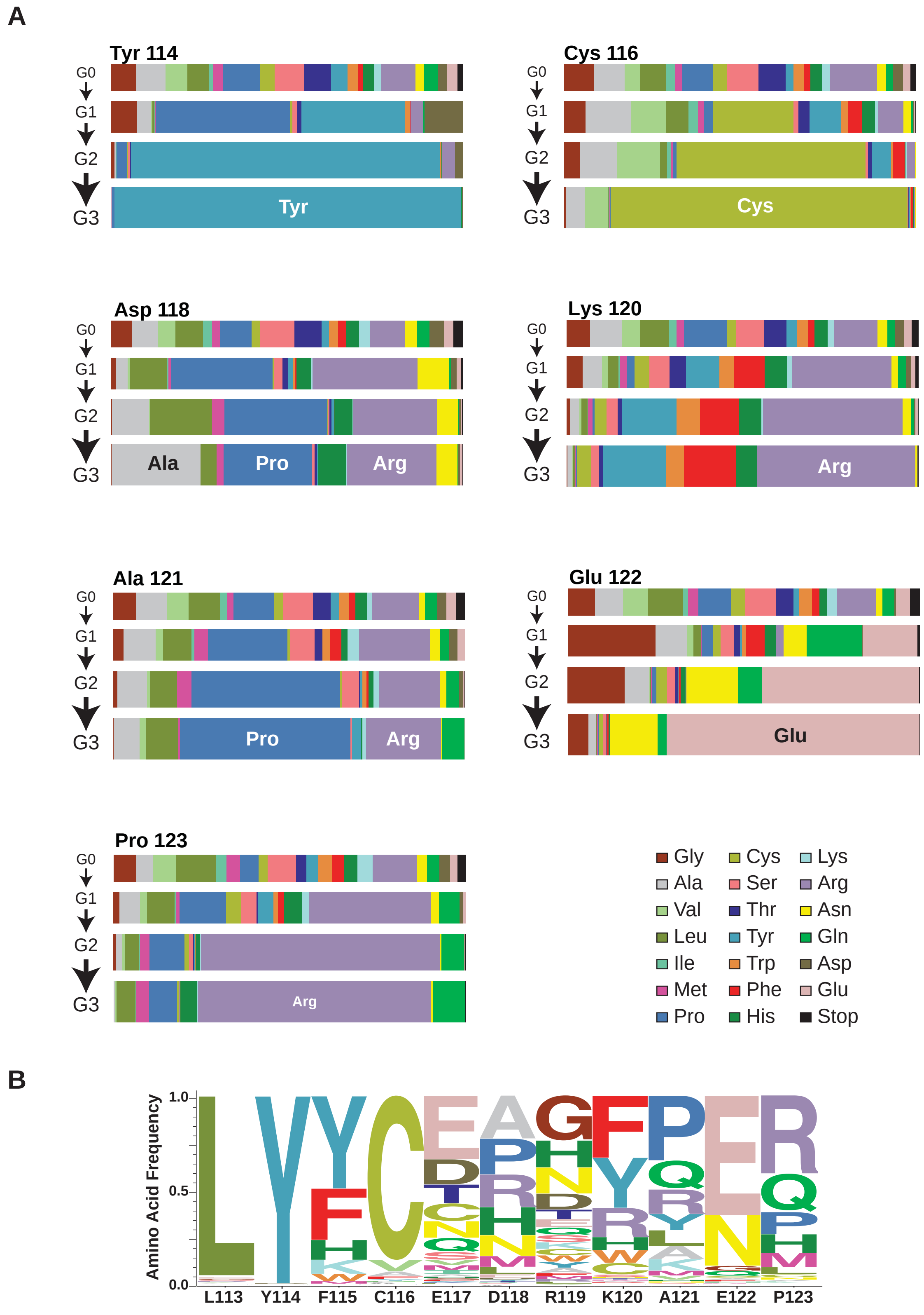

Figure S4. Functional determinants across the entire Leu113-Pro123 loop revealed by Sat-Sel-Seq. (**A**) Horizontal bar plots depicting the prevalence of amino acids from the four generational libraries, G0 to G3, for the positions not depicted in Figure 3. (**B**) Shown is a normalized logo plot for residues 113-123 summarizing the frequency of each amino acid at each position in the final selected library, adjusted for the prevalence of each amino acid in the G0 library. The corrected counts for each amino acid X in G3 were made by $G3^{X}_{Cor} = G3^{X} (G3^{X}/G3^{total})/(G0^{X}/G0^{total})$.

Euclidean Distance

	113	114	115	116	117	118	119	120	121	122	123	115 Alt
**113**		1.36	1.06	1.26	0.99	0.99	1.01	1.06	1.00	1.18	1.10	1.09
**114**	0.00		0.69	1.30	1.04	1.08	1.06	0.94	1.10	1.23	1.19	0.64
**115**	0.01	0.75		0.99	0.62	0.65	0.62	0.53	0.73	0.89	0.83	0.22
**116**	0.00	0.01	0.01		0.86	0.95	0.93	0.95	1.00	1.11	1.08	1.01
**117**	0.03	0.01	0.03	0.19		0.54	0.44	0.61	0.64	0.60	0.76	0.65
**118**	0.11	0.01	0.09	0.04	0.13		0.55	0.47	0.32	0.84	0.52	0.67
**119**	0.05	0.03	0.14	0.05	0.37	0.19		0.62	0.67	0.78	0.76	0.63
**120**	0.02	0.35	0.48	0.09	0.07	0.54	0.14		0.60	0.89	0.35	0.57
**121**	0.17	0.05	0.05	0.01	0.05	0.81	0.07	0.37		0.91	0.61	0.76
**122**	0.01	0.00	0.01	0.02	0.58	0.06	0.18	0.01	0.02		1.00	0.92
**123**	0.09	0.00	0.05	0.01	0.05	0.65	0.11	0.87	0.52	0.01		0.84
**115 Alt**	0.00	0.79	0.92	0.01	0.03	0.12	0.19	0.43	0.05	0.01	0.07	

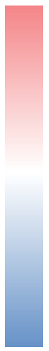

Cosine Similarity

$F_{n,x}$ = Frequency of amino acid n in G3 Library at Position x

Euclidean Distance between Position x and Position y results

$$\sqrt{\sum_{\substack{n = \text{all} \\ \text{amino acids}}} \left(F_{n,x} - F_{n,y}\right)^2}$$

Cosine Similarity between Position x and Position y results

$$\frac{\sum_{\substack{n = \text{all} \\ \text{amino acids}}} \left(F_{n,x} * F_{n,y}\right)}{\sqrt{\sum_{\substack{n = \text{all} \\ \text{amino acids}}} \left(F_{n,x}\right)^2} * \sqrt{\sum_{\substack{n = \text{all} \\ \text{amino acids}}} \left(F_{n,y}\right)^2}}$$

Figure S5. Similarity measures between positions. For each starting library, the G3 library was converted into a vector encoding the frequency ($F_{n,x}$) of each of the twenty amino acids (n) at that position (x). The distance between the library x and library y vectors was determined by two different metrics. Cosine similarity ranges between 0 (different) to 1 (identical). For the Euclidean distance, identical sequences have a metric of 0, while a larger metric means data are more dissimilar. The upper right of the table represents the Euclidean distance metric while the lower left of the table represents the cosine similarity, and entries are shaded from most identical (red) to most dissimilar (blue). The reproducibility of selection in the primary and alternative position 115 libraries is evident in the results.

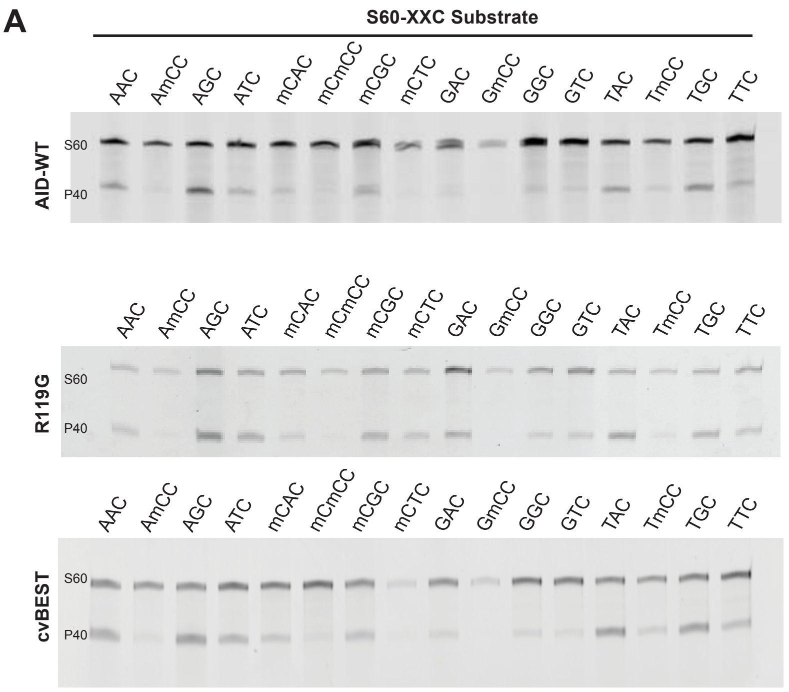

B

AID-WT

-2 position \ -1 position	A	mC	G	T
A	20	3	40	11
mC	6	1	13	6
G	4	2	3	2
T	21	9	30	9

R119G

-2 position \ -1 position	A	mC	G	T
A	45	11	65	30
mC	16	5	25	9
G	12	1	7	5
T	52	20	43	21

cvBEST

-2 position \ -1 position	A	mC	G	T
A	57	18	66	39
mC	23	6	38	27
G	20	11	12	9
T	54	28	45	25

Figure S6. Sequence Preference Profiles (**A**) To determine the sequence preference profiles, AID-WT, R119G and cvBEST were assayed against a panel of sixteen 3'-fluorophore labeled ssDNA 60 bp substrates containing a single cytosine in the context of varying -1 and -2 position residues (S60-XXC). The residue X was varied between A, 5-methylcytosine (mC), G and T. The reactions were run with 150 nM DNA for 3 hrs with enzyme concentrations selected such that substrate turnover was not complete under any condition (AID-WT 200 nM, R119G 80 nM, cvBEST 80 nM). The S60 substrates were fragmented to generate a 40 bp product by treatment with UDG and base. Shown is a representative gel from one assay. (**B**) Product formation from two replicates was averaged to give the percent product formed under standard conditions with each substrate, with associated error of <25% for each measurement. Sequence preference profiles (Figure 5B) were generated by a two-step calculation. First, the product formation was averaged for all sequences that contain the same nucleotide at either the -1 or -2 position. For example, P60-XAC (the average product formation for the S60-XAC substrates) was calculated by averaging product formation with S60-AAC, -mCAC, -GAC and -TAC. Next, the probability of deamination for each nucleotide at the -1 or -2 position was calculated relative to the other nucleotide variants. For example, the percent preference for A at -1 was calculated as (P60-XAC/(P60-XAC + P60-XmCC + P60-XGC + P60-XTC)) x 100.

SUPPLEMENTARY TABLES

Table S1 . Hydrogen Bonding Interactions Between AID and 5'-AGCT-3'.

Residue	AID-WT	R119G	cvBEST
**113**	-	GUA-Side-N2 LEU113-Main-O 15.07%<br>GUA-Side-N1 LEU113-Main-O 10.10%	GUA-Side-N2 LEU113-Main-O 30.95%<br>GUA-Side-N1 LEU113-Main-O 53.35%
**114**	-	-	GUA-Side-N2 TYR114-Main-O 3.03%
**117**	GUA-Side-N2 GLU117-Side-OE1 3.93%<br>GUA-Side-N2 GLU117-Side-OE2 3.68%<br>GUA-Side-N1 GLU117-Side-OE2 3.45%<br>GUA-Side-N1 GLU117-Side-OE1 2.40%	GUA-Side-N2 GLU117-Side-OE2 5.82%<br>GUA-Side-N2 GLU117-Side-OE1 4.83%<br>GUA-Side-N1 GLU117-Side-OE2 3.70%<br>GUA-Side-N1 GLU117-Side-OE1 3.18%	-
**119**	ARG119-Side-NH2 GUA-Side-N7 16.65%<br>ARG119-Side-NH1 GUA-Side-O6 9.48%<br>ARG119-Side-NE GUA-Side-O6 6.48%<br>ARG119-Side-NH2 ADE-Side-O5' 2.92%<br>ARG119-Side-NH1 GUA-Side-N7 2.85%<br>ARG119-Side-NH2 GUA-Side-O2P 2.27%	-	-

Hydrogen bond occupancy analysis was performed using HBonds Plugin (Version 1.2) in VMD. The hydrogen bond occupancy reflects the percentage of a simulation that a particular hydrogen bond exists with given cutoff criteria. Moderate and strong hydrogen bonds were included by defining a bond cutoff length of 3.2 Å (between centers of heavy atoms) and cutoff angle of >150 degrees. Listed are the hydrogen bonding interactions that occurred at > 2% frequency during the simulation.

Table S2 . Solvent accessible surface area for side chain residues

Residue	Side Chain Size, Å^2	AID-WT, Å^2 (% Accessible)	Y114F, Å^2 (% Accessible)	R119G, Å^2 (% Accessible)	cvBEST, Å^2 (% Accessible)
113	163.7	36.9 (22.5%)	-	14.2 (8.7%)	35.0 (21.4%)
114	190.6 (F) 209.6 (Y)	116.5 (55.6%)	73.2 (38.4%)	-	-
115	196.7	5.6 (2.9%)	-	-	-

Solvent accessible surface area (SASA) for Residues 113, 114 and 115 were computed in VMD using a probe with radius of 1.4 Å. Reported values represent the average solvent exposed surface area of a given residue side chain over the NVT trajectory (in Å) or are scaled (for % Accessible) relative to the average total surface area of the residue (solvent exposed area plus buried surface area).

SUPPLEMENTARY VIDEO

Video S1. Molecular Dynamics Simulations of AID with 5'-AGCT-3' DNA. The AID model is shown in gray with highlighted residues L113 (yellow), Y114 (green), F115 (purple) and R119 (blue). The DNA is shaded from red to white in the 5' to 3' direction. The model was equilibrated for 40 ns with constrained DNA. Shown are the subsequent 120 ns of the simulation between AID-WT and unconstrained DNA.
